# P061. Clinical characteristics of elderly with headache in an outpatient geriatric setting in Italy

**DOI:** 10.1186/1129-2377-16-S1-A77

**Published:** 2015-09-28

**Authors:** Manuel Soldato, Maurizio Evangelista

**Affiliations:** Geriatric Service, Complesso Integrato Columbus, Association Columbus, Rome, Italy; Pain Therapy Unit, Istituto di Anestesiologia e Rianimazione, Università Cattolica del S. Cuore, Policlinico A, Gemelli, Rome, Italy

## Background

Multiple epidemiological studies show that headache is less prevalent in elderly people. Despite this, there is evidence that headache has a significant impact in elderly quality of life. There are few studies evaluating characteristics of elderly patients with headache. Aim of the present observational study was to evaluate the characteristics of elderly patients reporting headache in an outpatient geriatric service in Italy.

## Methods

Data were collected from October 2014 to March 2015 in patients over 70 years of age. We used the Pain Detect Scale, a previous validated instrument, to investigate presence of any type of non cancer pain. The scale detected presence of pain, including headache, and assessed intensity of pain during the last month, using a numeric rating scale. We also collected data about comorbidity, number of drugs taken and cognitive status (using MMSE score).

## Results

Mean age of 94 participants was 81 years, 65 (69.1%) were women and 29 (30.8%) were men. Eight (8.5%) patients reported headache with significant pain intensity (VAS > 5). Patients with headache had high comorbidity (mean of 6 different diagnoses) and took multiple drugs (mean of 6 different drugs). Two of the 8 patients had a significant cognitive decline (MMSE < 23) (Table 1).

**Table 1:** Clinical characteristics of elderly with headache.

PATIENT ID	SEX	AGE	N. OF DRUGS	N. OF DISEASES	MMSE SCORE
**13**	F	70	11	7	30
**20**	F	81	4	5	28
**23**	F	78	6	11	16
**49**	M	75	5	6	27
**66**	M	76	4	5	25
**79**	F	82	10	5	27
**89**	F	75	3	4	30
**93**	F	80	7	6	21

## Conclusions

Among older adults assisted in outpatient setting, headache is not highly prevalent but when present, is a cause of significant chronic pain. Elderly persons with headache are a frail population with high comorbidity and multiple drug therapies that can represent a challenge for physicians involved in pain management.

Written informed consent to publication was obtained from the patient(s).

